# Effect of human T-cell lymphotrophic virus type 1 (HTLV-1) in seropositive infertile women on intracytoplasmic sperm injection (ICSI) outcome

**Published:** 2014-01

**Authors:** Mahnaz Mansouri Torshizi, Amir Reza Khalighi, Mahla Fadavi Islam, Rahele Aram, Elham Sabouri, Hekmat Khalilifar, Hesam Roustaee

**Affiliations:** 1 *Novin Infertility Treatment Center, Mashhad, Iran.*; 2 *Mashhad University of Medical Sciences, Mashhad, Iran.*

**Keywords:** *HTLV-1*, *ICSI outcome*, *Seropositive*, *Infertile*

## Abstract

**Background:** Human T-cell Lymphotrophic virus type 1 (HTLV-1) has infected more than 20 million people worldwide. Northeast of Iran, Mashhad, the capital of Razavi Khorasan Province, is endemic for HTLV-1 with a prevalence of 3% among general population.

**Objective: **We evaluated the ICSI outcome in our program for (HTLV-1) serodiscordant couples (SDCs) with the female infected in comparison with control group.

**Materials and Methods:** This study was performed between 2007 and 2011 in Novin Infertility Treatment Center (Mashhad, Iran). We examined 32 ICSI cycles of HTLV-1 infected women in comparison with an age matched control group (n=62). ICSI outcome was compared regarding fertilization rate (FR), embryo quality parameters, implantation rate (IR), clinical pregnancy rate (PR), and abortion rate (AR).

**Results:** Fertilization (p=0.15), implantation (p=0.33), and pregnancy rate (p=0.12) were similar between the groups. No difference was found regarding the number of transferred embryos (on day 2 or 3) and cryopreserved embryos, multiple pregnancies, or abortion rates between the groups.

**Conclusion:** Our results suggest that the embryo quality and ICSI outcome are not affected by HTLV-1 infection in serodiscordant couples. The major finding of this study is that the outcome of ICSI in HIV-I-infected patients and seronegative controls is similar.

## Introduction

Human T-cell lymphotropic virus type 1 (HTLV-1) was the first human retrovirus to be described. It was discovered simultaneously in the United States and in Japan in 1980. As documented for all retroviruses, HTLV-1 produces a permanent cell infection. Therefore, all carriers are potential sources of transmission of the infection (1). A Delta type of *Retroviridae virus *family called HTLV-1 has infected more than 20 million people worldwide (2). Endemic locations for HTLV-1 have prevalence ratio of over 1%, including Caribbean Basin, Central Africa, and South Japan (3). 

A nationwide endemic location for HTLV-1 in Iran is Mashhad, the capital of Khorasan Razavi province at northeast of Iran with prevalence ratio of above 3% (4-7). To our knowledge, there have been no previous reports describing assisted reproduction protocols exclusively in HTLV-1-positive patients. On the other hand HTLV-1,2 transmission occurs by mother to child and sexual contacts (8, 9).

HTLV-1can also cause inflammation of the eye (uveitis), joints (arthritis), muscles (myositis), lung (alveolitis), and skin (dermatitis). These conditions are even less common than ATLL (Adult T-cell Leukaemia/ Lymphoma) and HAM (HTLV-1-associated myelopathy) and the skin condition is usually only seen in tropical climates. There are several arguments indicating that HTLV-1 is a STD (sexually transmitted diseases) in Latin America. The virus has been found in semen and cervical secretions of infected people and sexual intercourse is an important factor for HTLV-1 transmission (10). Male-female sexual transmission is more efficient than female-male transmission (11).

## Materials and methods

A non-random sampling and a cross-sectional descriptive-analytic study, was performed between September 2007 and August 2011 at Novin Infertility Treatment Center (Mashhad, Iran). We examined 32 ICSI cycles of HTLV-1 infected women in comparison with an age matched control group (n=62). This study was not blind. The study was approved by the ethical committee of Infertility center and patients signed consent to participate in the study. A general viral screening was performed on couples before attempting ART including HIV, HTLV-1, hepatitis B, and hepatitis C viruses. After testing the blood samples by ELISA, Western blot (WB) analysis (Genelab Kit) was used for confirmation of positive HTLV-1 associated ELISA results. 

Criteria for HTLV-1 seropositivity in PCR test was reactivity to GAG proteins (p19 with or without p24). The age of women ranged from 29-46 years old. The control group (62 couples) was matched with regard to age and oocyte retrieval period. The standard long protocol was adopted for most ovulation stimulation cycles. The short protocol was used for patients who were poor responders. Both groups were compared regarding cycle parameters (gonadotropin dose, stimulation days, number of total and mature oocytes per retrieval). 

Moreover, ICSI outcome was compared regarding fertilization rate (FR), embryo quality parameters, implantation rate (IR), clinical pregnancy rate (PR), and abortion rate (AR) (12, 13). On the other hand, male infertility parameters and semen quality were compared between experimental and control group. Semen samples were obtained by masturbation and then tested for sperm concentrations, motility, and normal morphology. Sperm quality was reported according to the World Health Organization guidelines (14). An improved swim up method was used to washing the sperm samples. 

Then, all recovered metaphase II oocytes were microinjected. Oocyte fertilization was assessed at 16-20 hours post-ICSI. Embryo morphology was evaluated on days 2 or 3 regarding the number, symmetry, and granularity of blastomeres, percentage of fragmentation, and compaction degree. According to the symmetry of the blastomeres and fragmentation, we defined three types of embryos: 1) equal-size blastomeres with no fragmentation; 2) unequal-size blastomeres with <20% fragmentation; and 3) evidence of >50% fragmentation (15).

**Statistical analysis**

Statical analysis was performed by using the statistical package for the Social Science (SPSS 19) .Either t test and chi-square were used to test differences between HTLV-1 serodiscordant couples and control couples. Significance was defined at p<0.05.

## Results

In this study, half of our infected population was aged >35 years. In fact, the mean age of our HTLV-1-infected patients was higher than the other patients undergoing ICSI at our facility. Because of this difference, we compared the outcome of ICSI obtained in HTLV-1-infected women (group I) to those obtained in a matched control group (p<0.05). Controls were matched with regard to age and oocyte retrieval period. The patients’ characteristics are summarized in table I. 

The ICSI outcomes compared in the 32 HTLV-1 serodiscordant and 62 healthy seronegative couples (control group). The fertilization and development of about 1199 oocytes were analyzed in this study, which represented 12.9±6.9 and 12.6±7.6 oocytes retrieved per couple (serodiscordant and control group, respectively). Of these, 11.31±5.8 and 10.7±6.5 oocyte per cycle were mature (serodiscordant and control group, respectively). There was no significant difference between two groups regarding to the cycle parameters (gonadotropin dose, stimulation days and the number of total and mature oocytes per retrieval). 

The number of transferred embryos (on day 2 or 3, 185 embryos transferred on day 2 and 187 embryo transferred on third day), fertilization, implantation, pregnancy, and abortion rate were also similar between two groups. In addition, on day 2 or 3, the parameters of embryo quality (cell number, equal-size of blastomeres, and fragmentation) were equal in both groups (Table II).

**Table I:** Characteristics of 32 serodiscordant couples and 62 control couples undergoing ICSI

	**SDC**	**Control**	
Number of couples	32	62	
Female age [years (Mean±SD)	35.8 ± 5.9	33.7 ± 5.5	0.42
Male age [years (Mean±SD)]	42 ± 8.1	37.4 ± 7.2	0.87
Sperm total count, (106/mL)(min-max)	32.7- 37.6	29.9- 33.09	
Motility of sperm	55	60	
Immotile sperm	15	15	
Normal morphology of sperm(mean)	75	80	

SDC: serodiscordant couple cycles

**Table II:** Outcome of ICSI in serodiscordant couple cycles (SDC) and control couples cycles

**Parameters**	**SDC**	**Control**	**p-value**
No. of cycles	32	62	
Gonadotropins dose, (Mean ± SD)	2296.09 ± 302.6	2255 ± 456.9	0.61
Stimulation days, (Mean ± SD)	10.01 ± 1.2	10.03 ± 1.02	0.83
No. of oocytes retrieved	415	784	
No. of mature oocytes retrieved	362	662	
No. of oocytes retrieved/couple	12.9 ± 6.9	12.6 ± 7.6	0.74
No. of mature oocytes retrieved/Couple	11.31 ± 5.8	10.7 ± 6.5	0.43
Fertilization rate, (%)	65	73	0.15
No. of embryos transferred	2.9 ± 0.9	2.8 ± 0.7	0.79
No. of embryos cryopreserved	4.4 ± 3.9	4.5 ± 4.3	0.68
Implantation rate, %	22.6	18.4	0.33
Pregnancy rate (%)	15.32 (46%)	28.62 (45%)	0.12
Abortion/cycle (%)	3.15 (20%)	5.28 (17%)	0.21
Multiple PR (%)	1.15 (6%)	3.28 (10%)	0.09

PR: Pregnancy rate.

Student t-test and chi-square test.

## Discussion

HTLV-1 was the first retrovirus linked to human disease. It has been convincingly associated with ATLL (adult T-cell leukemia/ lymphoma), HAM/TSP, uveitis. HTLV-1 has also been linked to cases of polymyositis, synovitis, thyroiditis and bronchio-alveolar pneumonitis, although definitive epidemiologic proof of HTLV-1 association is lacking. The two major HTLV-1-associated diseases, ATLL and HAM/TSP, are present in all endemic areas, although prevalence and incidence rates show significant geographic heterogeneity (16, 17). Common ways of infection and modes of transmission for HTLV-1 are found among human normal interactions like mother to child transmission due to pregnancy or breast feeding, sex and invasive process like infusion of infected blood or its products or sharing needles and syringes (18). 

To date, no report is found in the specialized literature regarding the ICSI outcome such as embryo quality, fertilization rate, implantation rate, pregnancy rate and etc. in HTLV-1 seropositive female. To date, all studies including cycles on HTLV-1 infection have mainly focused on decreasing the risk of transmission. In addition some papers indicated that HTLV-1 causes no disorders in a majority of healthy asymptomatic virus carriers (19, 20). So this is the first study to evaluate the outcome of ART in HTLV-1 serodiscordant couples. It was clarified that the effectiveness rates for successful pregnancy are similar to those of the general population with equivalent infertility and age profiles. 

To assess the possible effects of HTLV-1 on the reproduction system and sexual cells of couples we decided to study the relation between HTLV-1 serodiscordant with HTLV-1 seronegative couples in ICSI outcomes. Currently because of the lack of serologic tests to differentiate HTLV-2 from HTLV-1, there is no information available regarding to the seroepidemiology or modes of transmission of HTLV-1,2 in assistant reproductive technology (ART). There was not remarkable number of HTLV-1 and 2 seropositive couples among our patients who voluntaries for intracytoplasmic sperm injection (ICSI) program to dividing them in two groups, so that we just consider on group (HTLV-1 seropositive) study group. 

As a result in this study, no statistically differences were seen between the fertilization rate and the quality of obtained embryos days 2 or 3 in two groups. In this study, our results suggest that the embryo quality and ICSI outcome are not affected by HTLV-1 infection in serodiscordant couples. The major finding of this study is that the outcome of ICSI in HIV-1-infected patients and seronegative controls is similar.

## References

[1] Gotuzzo E, Verdonck K (2004). HTLV-1: clinical impact of a chronic infection. The infectious etiology of chronic diseases: defining the relationship, enhancing the research, and mitigating the effects.

[2] Proietti FA, Carneiro-Proietti AB, Catalan-Soares BC, Murphy EL (2005). Global epidemiology of HTLV-1 infectionand associated diseases. Oncogene.

[3] Vrielink H, Reesink HW (2004). HTLV-I/II prevalence in different geographic locations. Transfus Med Rev.

[4] Hedayati-Moghaddam MR, Fathimoghadam F, Eftekharzadeh Mashhadi I, Soghandi L, Bidkhori HR (2011). Epidemiology of HTLV-1 in Neyshabour, Northeast of Iran. Iran Red Crescent Med J.

[5] Kasper DL, Braunwald E, Fauci AS, Hauser SL, Longo DL, Jameson JL (2008). Harrison's principles of internal medicine.

[6] Mandell GL, Bennett JE, Dolin R. Mandell, Douglas (2010). Bennett's Principles and Practice of Infectious Diseases.

[7] Safai B, Huang JL, Boeri E, Farid R, Raffat J, Schutzer P (1996). Prevalence of HTLV type I infection in Iran: a serological and genetic study. AIDS ResHum Retroviruses.

[8] Lal RB, Brodine SK, Coligan JE, Roberts CR (1991). Differential antibody responsiveness to p19 gag results in serological discrimination between human T-lymphotropic virus type I and type II. J Med Virol.

[9] Chen YM, Lee TH, Wiktor SZ, Shaw GM, Murphy EL, Blattner WA (1990). Type-specific antigens for serological discrimination of HTLV-I and HTLV-II infection. Lancet.

[10] Beilke MA, Traina-Dorge V, England JD, Blanchard JL (1996). Polymyositis, arthritis, and uveitis in a macaque experimentally infected with human T lymphotropic virus type I. Arthritis Rheum.

[11] Glynn JR, Caraël M, Auvert B, Kahindo M, Chege J, Musonda R Why do young women have a much higher prevalence of HIV than young men?. AIDS.

[12] TerriouP, AuquierV, Chabert-OrsiniJM, ChincholeL, CravelloC, Giorgetti P (2005). Outcome of ICSI in HIV-1-infected women. Hum Reprod.

[13] Barreto Melo MA, Meseguer M, Bellver J, Remoh J, Pellicer A, Garrido N (2008). Human immunodeficiency type-1 virus (HIV-1) infection in serodiscordant couples (SDCs) does not have an impact on embryo quality or intracytoplasmic sperm injection (ICSI) outcome. Fertil Steril.

[14] Cooper TG, Noonan E, von Eckardstein S, Auger J, Baker HWG, Behre HM (2010). World Health Organization reference values for human semen characteristics. Hum Reprod Update.

[15] Racowsky C, Stern JE, Gibbons WE, Behr B, Pomeroy KO, Biggers JD (2011). National collection of embryo morphology data into Society for Assisted Reproductive Technology Clinic Outcomes Reporting System: associations among day 3 cell number, fragmentation and blastomere asymmetry, and live birth rate. Fertil Steril.

[16] Proietti FA, Carneiro-Proietti AB, Catalan-Soares BC, Murphy EL (2005). Global epidemiology of HTLV-1 infection and associated diseases. Oncogene.

[17] Poiesz BJ, Ruscetti FW, Gazdar AF, Bunn PA, Minna JD, Gallo RC (1980). Detection and isolation of type C retrovirus particles from fresh and cultured lymphocytes of a patient with cutaneous T-cell lymphoma. Proc Natl Acad Sci USA.

[18] Manns A, Hisada M, La Grenade L (1999). Human T-lymphotropic virus type I infection. Lancet.

[19] Fujino T, Nagata Y (2000). HTLV-1 transmission from mother to child. J Reprod Immunol.

[20] Ho GYF, Kenrad N, Nomura AMY, Polk BF, Blattner WA (1992). Markers of Health Status in an HTLV-1- Positive Cohort. Am J Epidemiol.

[21] LaGrenade L, Hanchard B, Fletcher V, Cranston B, Blattner W (1990). Infective dermatitis of Jamaican children: a marker for HTLV-1 infection. Lancet.

[22] Sabouri AH, Usuku K, Hayashi D, Izumo S, Ohara Y, Osame M (2008). Impaired function of human T-lymphotropic virus type 1 (HTLV-1)- specific CD8+ T cells in HTLV-1-associated neurologic disease. Blood.

[23] CDC (Centers for Disease Control and Prevention) (1993). Recommendations for counseling persons infected with human T-lymphotropic virus, types I and II. Recommendations on prophylaxis and therapy for disseminated Mycobacterium avium complex for adults and adolescents infected with human immunodeficiency virus. MMWR.

[24] Benifla JL, Letur-Konïrsch H, Collin G, Devaux A, Kuttenn F, Madelenat P (2000). Safety of cryopreservation straws for human gametes or embryos: a preliminary study with human immunodeficiency virus-1. Hum Reprod.

[25] Kushnir VA, Lewis W (2011). Human immunodeficiency virus/acquired immunodeficiency syndrome and infertility: emerging problems in the era of highly active antiretrovirals. Fertil Steril.

[26] Englert Y, Van Vooren JP, Place I, Liesnard C, Laruelle C, Delbaere A (2001). ART in HIV-infected couples has the time come for a change of attitude?. Hum Reprod.

[27] Savasi V, Mandia L, Laoreti A, Cetin I (2013). Reproductive assistance in HIV serodiscordant couples Hum. Hum Reprod Update.

[28] Vandermaelen A, Englert Y (2010). Human immunodeficiency virus serodiscordant couples on highly active antiretroviral therapies with undetectable viral load: conception by unprotected sexual intercourse or by assisted reproduction techniques?. Hum Reprod.

